# Beyond lacunae: Dermoscopic features of angiomas

**DOI:** 10.1016/j.jdin.2026.03.002

**Published:** 2026-03-13

**Authors:** Sebastián González-Valdés, Sofia Villagrán-Essmann, Marie-Chantal Caussade, Cristian Navarrete-Dechent, Alvaro Abarzúa-Araya

**Affiliations:** a Department of Dermatology, Escuela de Medicina, Pontificia Universidad Católica de Chile, Santiago, Chile; b Melanoma and Skin Cancer Unit, Escuela de Medicina, Pontificia Universidad Católica de Chile, Santiago, Chile

**Keywords:** angioma, dermoscopy, dermatoscopy, melanoma, skin cancer, vascular

*To the Editor:* Angiomas are common benign vascular tumors in adults.^1^ Under dermoscopy, they show lacunae and septa,^2^ yet advances in dermoscopy reveal additional vascular structures; however, these have not been extensively characterized.^3^ Importantly, angiomas may mimic melanoma metastases with an angioma-like pattern, underscoring the need for detailed dermoscopic characterization.^4^ Our objective was to describe dermoscopic findings in angiomas and associations with patient characteristics.

We conducted an institutional review board-approved cross-sectional study at a tertiary hospital in Santiago, Chile (September 2023-June 2024). Consecutive adults presenting for routine full skin examinations with ≥2 angiomas were included; patients with a history of prior invasive melanoma were excluded. Patients were followed up at least once more within 1 year. Two to five trunk angiomas per patient were randomly selected and photographed. Clinical and dermoscopic images were reviewed by 2 investigators (S.G-V. and S.V-E.) for consensus; disagreements were resolved by a third investigator. We recorded the following dermoscopic criteria: septa, lacunae, linear irregular vessels, corkscrew vessels, and comma vessels. Additionally, we defined a new term, multi-looped vessels, which consists of a group of well-defined vessels forming multiple loops that do not fit in other vessel category. To account for clustering of multiple lesions within individual patients, associations were evaluated using generalized estimating equation models. Statistical analyses were conducted using IBM SPSS26.0.

A total of 380 lesions in 99 consecutive patients (45.5% females; mean age 63.3 ± 15.3 years) were included. The mean lesion size was 2.20 ± 1.14 mm. Dermoscopy showed lacunae in 68.7%, multi-looped vessels in 64.5%, septa in 37.4%, linear irregular vessels in 30.3%, comma vessels in 2.4%, and corkscrew vessels in 1.3% of lesions **(**Supplementary Figs 1 and 2, https://data.mendeley.com/datasets/dvmkyv8yds/1). Lesions with presence of lacunae and septa had a larger mean size compared with those without (lacunae 2.50 ± 1.18 vs 1.55 ± 0.69 mm; *P* < .001 and septa 3.17 ± 1.09 vs 1.62 ± 0.68 mm; *P* < .001). Lesions with multi-looped and linear irregular vessels had a smaller mean size compared with those without (multi-looped 1.96 ± 0.88 vs 2.64 ± 1.40 mm; *P* < .001 and linear irregular 1.91 ± 0.83 vs 2.33 ± 1.23 mm; *P* = .001). Additional associations are shown in Table I.

**Table I:** Patient demographics, clinical features, and associations of vessel types and septa with lesion size and age

Parameter	Lesions (*n* = 380)
No. of patients	*N* = 99
Female sex, *n* (%)	45 (45.5%)
Age, mean ± SD, y	63.3 ± 15.3
Location of lesions, *n* (%)	
Anterior trunk	288 (75.8%)
Posterior trunk	92 (24.2%)
Size, mean ± SD (range), mm	2.20 ± 1.14 (0.5-7)
Dermoscopic features, *n* (%)	
Lacunae	261 (68.7%)
Multi-looped vessels	245 (64.5%)
Linear irregular vessels	115 (30.3%)
Comma-shaped vessels	9 (2.4%)
Corkscrew vessels	5 (1.3%)
Septa	142 (37.4%)

Characteristic			*P*∗	OR (95% CI)∗
Lesion size, mean ± SD, mm	Presence	Absence		
Lacunae	2.50 ± 1.18	1.55 ± 0.69	< .001	3.15 (2.19-4.55)
Multi-looped vessels	1.96 ± 0.88	2.64 ± 1.40	< .001	0.62 (0.50-0.76)
Linear irregular vessels	1.91 ± 0.83	2.33 ± 1.23	.001	0.71 (0.58-0.87)
Septa	3.17 ± 1.09	1.62 ± 0.68	< .001	11.39 (6.61-19.62)
Patient age, mean ± SD, y	Presence	Absence		
Lacunae	63.70 ± 15.16	61.92 ± 15.18	0.449	1.07 (0.89-1.29)
Multi-looped vessels	62.14 ± 15.01	64.97 ± 15.33	0.180	0.88 (0.74-1.06)
Linear irregular vessels	62.39 ± 16.18	63.47 ± 14.72	0.501	0.95 (0.82-1.10)
Septa	65.58 ± 13.45	61.69 ± 15.95	0.048	1.20 (1.00-1.44)

*n*, Absolute number; *OR*, odds ratio.

∗ All values were calculated using generalized estimating equations.

Herein, we suggest that the presence of polymorphous vessels is a common finding in angiomas: Classic lacunae and septa predominate in larger lesions, whereas smaller lesions mainly show multi-looped and linear irregular vessels. Based on these findings, we propose a hypothetical progression model from an initial angioma to a mature one (Fig 1).

**Fig 1:**
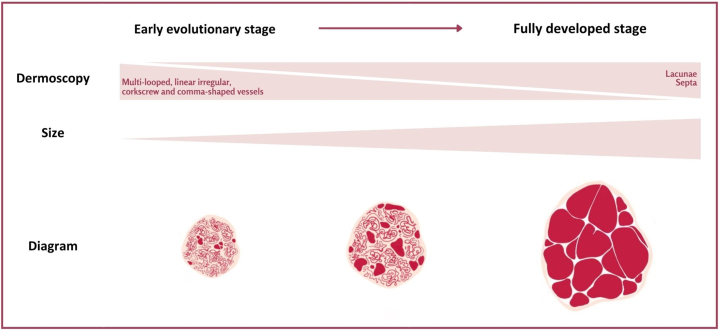
Illustration of the proposed model of progression from initial angioma to mature angioma.

Limitations include lack of a control group and histopathological confirmation; however, dermoscopy is known to have high specificity for diagnosing angiomas making misdiagnosis unlikely.^5^ To minimize diagnostic uncertainty and overlap with melanoma metastasis, patients were followed regularly, with no cases of melanoma observed during follow-up. Patients with a history of invasive melanoma were excluded.

This study highlights the types of vessels seen in angiomas under dermoscopy and their associations. Future prospective studies could complement these findings.

## Conflicts of interest

None disclosed.
